# Garnet U-Pb and O isotopic determinations reveal a shear-zone induced hydrothermal system

**DOI:** 10.1038/s41598-019-46868-4

**Published:** 2019-07-17

**Authors:** Zhongjiang Zang, Leilei Dong, Wei Liu, Han Zhao, Xinshui Wang, Keda Cai, Bo Wan

**Affiliations:** 1 State Key Laboratory of Lithospheric Evolution, Institute of Geology and Geophysics, Chinese Academy of Sciences, Beijing, 100029 China; 2 College of Earth and Planetary Sciences, University of Chinese Academy of Sciences, Beijing, 100049 China; 3 Faculty of Earth Resources, China University of Geosciences, Wuhan, 430074 China; 4 Key Laboratory of Deep-Earth Dynamics, Institute of Geology, Chinese Academy of Geological Sciences, Beijing, 100037 China; 5 Key Laboratory of Mineral Resources, Institute of Geology and Geophysics, Chinese Academy of Sciences, Beijing, 100029 China; 6 State Key Laboratory of Geological Processes and Mineral Resources, and School of Earth Science and Resources, China University of Geosciences, Beijing, 100083 China

**Keywords:** Geochemistry, Mineralogy

## Abstract

The absolute crystallization ages of minerals from hydrothermal fluids measured *in situ* can unravel the timing of key events leading to the formation of, for instance, ore deposits and hydrothermally derived geological terrains. In this study, a skarn iron deposit from northwest (NW) China is shown to have U-Pb garnet and U-Pb zircon ages of 254.2 ± 1.7 Ma and 255.5 ± 1.0 Ma, respectively, which are both significantly younger than magmatism and metamorphism of the region. This skarn age instead correlates with the occurrence of strike-slip and thrust faulting in the region. The water/rock mass ratio of 0.065~0.115 suggests the δ^18^O garnet composition is ~1‰ at temperatures ranging from 250–450 °C. The low oxygen isotopic composition indicates the role of meteoric water in the garnet formation. These measurements can be interpreted as the shear along faults circulating meteoric water ~10 km below the hanging wall of meta-volcanic sedimentary rock. Meteoric water in this hydrothermal system would leach cations from the meta-volcano-sedimentary rocks necessary for mineralization. Silica-rich hydrothermal fluid reacts with calcic-rich materials in the meta-volcano-sedimentary rocks, depositing the garnet and magnetite. Our work suggests that the shear zone is rich in ores, rendering this deposit for NW China a prospective source for future mineral resource exploration.

## Introduction

Garnet is an abundant mineral phase found in a range geological settings, from skarn-type ore deposits, granite, and low- and high-grade metamorphic rocks, to the upper mantle. It is a widely used proxy in geothermobarometry^1,2^. The stability and generally porphyroblastic nature of garnet makes it an ideal mineral for tracking the evolution of crystallization by measuring the elemental and isotopic compositions of different crystallization zones in garnet^3^. On the other hand, heavy rare-earth elements are compatible in garnet, which can provide absolute age constraints using, for instance, the Lu-Hf and Sm-Nd isotopic systems^4,5^. More recently, with the advancement of the spatial and temporal resolution of laser ablation inductively coupled plasma mass spectrometry (LA-ICP-MS), *in-situ* analyses of U-Pb isotopes on magmatic-hydrothermal garnet have successfully determined concordia ages that are identical to magmatic zircon U-Pb ages^6–9^. Notably, detailed U mapping of garnet illustrates a uniform distribution, indicating that U occurs within the garnet crystal lattice^10^. U-Pb ages of garnet therefore represent crystallization ages of the rock.

**Figure 1:**
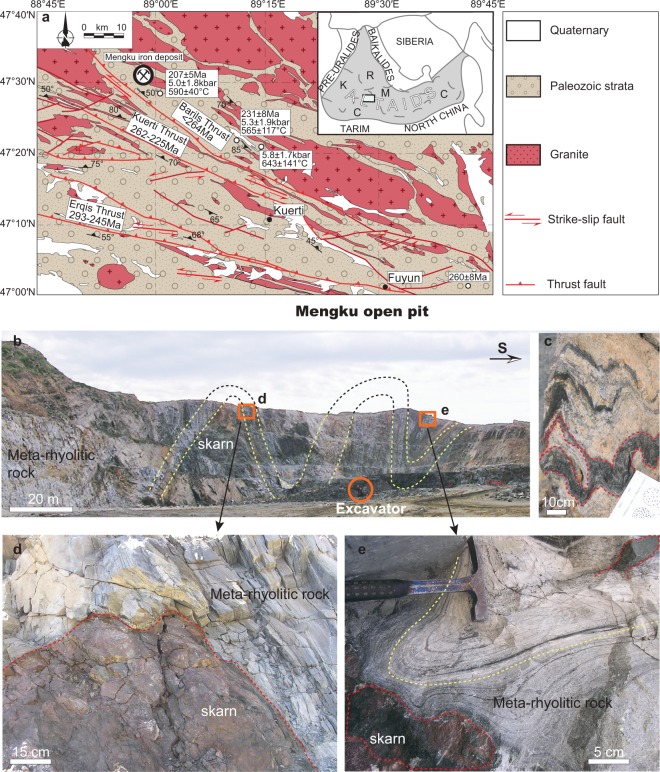
(**a**) The geological map of the Mengku iron deposit in the Fuyun. Small black open circles with the age value, pressure, temperature are the results of Briggs^13^. (**b**–**d**) show that the skarn mineralization is strata bounded by the deformed host rocks in different scales. (**e**) shows the skarn cut across the fold axis of the hosting rock, indicating post-deformation formation. The locations of (**d**,**e**) are marked in the (**b**).

The Mengku iron deposit is located in the central part of the late Paleozoic Chinese Altai orogen, one of the southern segments of the Altaids orogenic collage (Fig. 1a), and occurs in the Early Devonian Kangbutiebao Formation^11^. Studies in the past decade have provided clear magmatic, structural, and metamorphic frameworks, improving our understanding of the geodynamic evolution of the Chinese Altai orogen^12–17^. The Kangbutiebao Formation is mainly composed of thick layers of meta-rhyolitic volcanoclastic rocks interlayered with thin layers of meta-sandstone and marble, all of which underwent greenschist to amphibolite facies metamorphism^18^. Detailed metamorphic studies on the formation have identified an early Barrovian event and two later Buchan events during the mid-Devonian to early Permian. Structural analyses indicate that the formation underwent early Carboniferous NE-SW trending upright folding, followed by early Permian NW-SE folding^19^. The Erqis shear zone is one of the largest transcurrent fault systems in Asia^12^. It is located at the southern footwall of the Kangbutiebao Formation. Structural and chronological studies have revealed that the Erqis shear zone caused a series of south-vergent thrusts and strike-slip faults across the Chinese Altai orogen during late Permian to Triassic time, similar to the Barils Thrust in the Kangbutiebao Formation^13,20^.

The Mengku ore deposit is located at the hanging wall of the Barils Thrust. Orebodies are lenticular and enclosed by skarn (Fig. 1b,c). All orebodies are stratabound to the Kangbutiebao Formation. The distribution of skarn and orebodies thus follows the folded strata at different scales (Fig. 1b,d). However, in this study, a skarn has been observed to cut across the fold axis of the hosting strata (Fig. 1e), indicating that the skarn and deposit should be younger than the folding. Mineralization probably formed in an early Devonian hydrothermal vent on the seafloor ^21^ or 400 Ma ± 6 Ma fluids of magmatic origin^22,23^. This is inconsistent with our previous and recent observations^11^. Previous Ar-Ar dates of biotite in marble from a plateau age of 221 ± 3 Ma suggest a thermal event relating to strike-slip fault movements^23^. U-Pb dating of hydrothermal zircon from skarn (250 ± 2 Ma)^11^ and Re-Os dating of a molybdenite-bearing quartz vein (261 ± 7 Ma)^23^ reveal that the hydrothermal fluid deposits in Mengku occurred in the late Permian to Triassic, which is younger than both the regional magmatic activity (450–270 Ma) and local granites in the mining area (400–378 Ma)^14,15,22–26^. Based on geological, chronological, and isotopic observations, we propose that meteoric water circulating in a shear zone triggered the Mengku iron mineralization^11^. However, previous chronological studies of the Mengku deposits are indirect constraints because the dated zircons and molybdenite are not linked with magnetite and skarn formations. The ore genesis of a skarn iron deposit can be described by the following equation^27^:

$$\begin{array}{lll}{\rm{calcite}}+{\rm{quartz}}+{\rm{Fe}}{({\rm{OH}})}_{{\rm{2}}}+{{\rm{FeCl}}}_{{\rm{2}}} & = & {\rm{diopsite}}+{\rm{andradite}}+{\rm{magnetite}}\\  &  & +\,{{\rm{CO}}}_{{\rm{2}}}+{\rm{HCl}}+{{\rm{H}}}_{{\rm{2}}}.\end{array}$$

**Figure 2:**
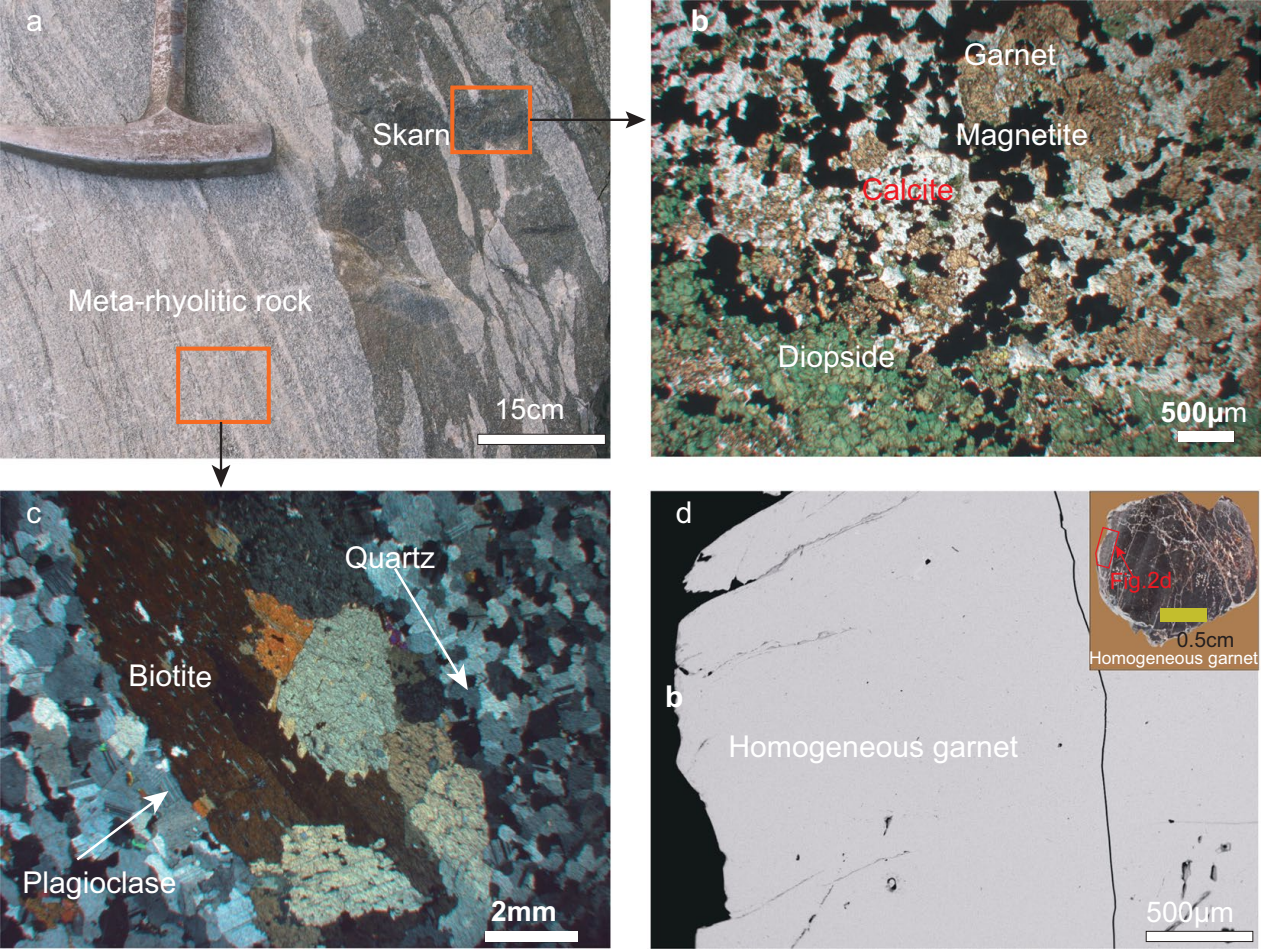
(**a**) The reaction relic texture of hydrothermal fluids and the hosting meta-rhyolitic volcanoclastic rocks. (**b**) typical skarn mineralization with garnet + diopside + magnetite + calcite. (**c**) shows orthogonal light plagioclase as polysynthetic twin. Quartz is recrystallized with elongated biotite. (**d**) Backscattered electron image of a homogenous garnet crystal. The BSE image location is marked in the large-garnet.

The skarn and magnetite are the principal products of metasomatic processes between silicic and calcic components. Such mineral assemblages have been observed in both outcrops and thin sections (Fig. 2a,b). Therefore, the age of the andradite indicates the age of iron mineralization. In the Mengku deposit, the marble formations could be the principal source of calcium. Furthermore, the meta-rhyolitic volcanoclastic rocks adjacent to the skarn contain feldspar, which could also supply some of the Ca to the skarn system (Fig. 2c). Quartz in Fig. 2c also recrystallized with the formation of biotite elongate with regional foliation, suggesting crustal depths of ~10 km, in line with the P-T estimates of the host rock^13^. The garnet in the Mengku deposit belongs to grandite^11^, bearing both zoned and non-zoned patterns. The flat compositional profile of garnet has an equilibrated composition, thus representing the average isotopic composition of the fluids. In this study, we conducted *in-situ* U-Pb analyses on a large, non-zoned garnet (Fig. 2d) in order to directly constrain the age of skarn formation and iron mineralization. In addition, oxygen isotopic measurements of the garnet were made to constrain the source of the fluids responsible for ore genesis of the skarn deposit.

## Results

EPMA data of the garnet and major elements in the host rocks are listed in Supplementary Dataset 1. The large garnet belongs to grandite and is mainly composed of ~80% andradite ~15% grossular, and ~5% of almandine. The meta-rhyolitic volcanoclastic host rock is enriched in Na_2_O (up to 8.05% wt.%) and depleted in CaO (low to 0.06% wt.%). Such a low content of Ca possibly indicates that the calcium was leached from the meta-rhyolitic rocks.

Oxygen isotope data are listed in Supplementary Dataset 2. Garnet powder for oxygen analyses by MAT252 gives δ^18^O_V-SMOW_ values of 1.12 ± 0.01‰.

**Figure 3:**
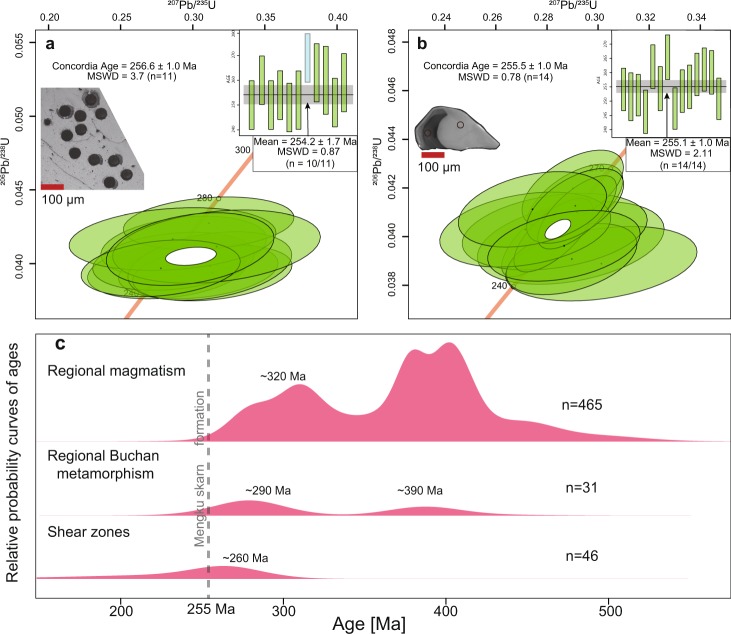
(**a**) The LA-ICP-MS U-Pb result of garnet with concordant and weighed mean ages. The blue bar was excluded from the calculation. (**b**) The Uranium-lead SIMS measurement of a representative zircon with concordant and weighed mean ages. Red circles show the location of geochronological points. (**c**) The age distributions of regional magmatism, metamorphism and shear activities in the Chinese Altai, n = number of ages from^14,15,22–26^.

LA-ICP-MS U-Pb analytical results of the large garnet (3 cm diameter) are listed in the Supplementary Dataset 3. A large garnet crystal measured for U-Pb from the iron-mineralized skarn is homogeneous without major element zoning as measured by EPMA. Eleven U-Pb spot analyses were made on randomly selected regions. The measured Pb/U ratios is concordant within analytical error, with a concordia age of 256.6 ± 1.0 Ma (MSWD = 3.7). The weighted mean ^206^Pb/^238^U age is 254.2 ± 1.7 Ma (MSWD = 0.87) (Fig. 3a).

Secondary ion mass spectrometry (SIMS) U-Pb measurements of zircons are listed in the Supplementary Dataset 3. The zircons were selected from the mineralized skarn (MK10). They are as large as 300 μm, displaying anhedral and resorption textures, with light and dark colored zones (Fig. 3b). Fourteen hydrothermal zircons from the iron-mineralized skarn were selected for U-Pb analyses. Th/U ratios are 0.15–0.33 (Supplementary Dataset 3). The measured Pb/U ratios are similar between different zircons and different zones in one zircon (255.5 ± 1.0 Ma, MSWD = 0.78). The weighted mean ^206^Pb/^238U^ age is 255.1 ± 1.0 Ma (MSWD = 2.11), which is interpreted as the crystallization time of the zircons (Fig. 3b).

## Discussion

### The timing of skarn formation and iron mineralization

Magnetite is the primary iron-bearing mineral that mostly occurs with diopside, andradite, quartz, calcite, and other calcic-silicate minerals (Fig. 2d). In the skarn formation, it is possible that iron-rich silicous fluid reacts with calcium-rich strata, producing a calcium-rich skarn and magnetite in the deformed strata. Therefore, the age of andradite directly constrains the iron mineralization. According to the *in-situ* LA-ICP-MS U-Pb result, the garnet formed at 254.2 ± 1.7 Ma (Fig. 3a), which is significantly younger than any outcrop of igneous rocks in the mining area. The timing of mineralization determined by SIMS of zircons from the mineralized skarn have a concordant age of 255.1 ± 1.0 Ma (Fig. 3b). The garnet U-Pb age is identical to the hydrothermal zircon U-Pb date. The garnet and zircon have been observed to occur as intergrowths in the Mengku deposit. The zircons are therefore interpreted as hydrothermal in origin. This is further substantiated by the occurrence of fluid- and hydrothermal-mineral inclusions^11^. In summary, the simultaneous crystallization of garnet and hydrothermal zircon are supported both by mineralogy and these geochronological results. We reported hydrothermal zircon ages of 250.2 ± 2 Ma from the mineralized skarn of the Mengku deposit with the LA-ICP-MS method^11^. Our previous result is slightly younger than the new results, but consistent within 2σ error. Consequently, formation of the Mengku iron deposit ~255 Ma ago is confirmed by the direct garnet U-Pb date.

The meta-rhyolitic volcanoclastics of the Kangbutiebao Formation in the Mengku ore deposit mainly formed at 404 ± 5 Ma^11^. The emplacement ages of the granites in the mine area range from 404 ± 8 Ma to 378 ± 7 Ma^22,24^. The structural geology of garnet-amphibolite schist 3-10 km southeast of the Mengku deposit is consistent with the thrusting movement of the Barils fault. The Th-Pb metamorphic ages of these rocks dated from monazite *in-situ* have an average age of 246 Ma ± 18 Ma, which is proposed as the constraint for the timing of regional shear movement^13^. The Mengku mineralization time is much younger than the magmatic ages in the mining area, but is consistent with the timing of metamorphism. If we compare the magmatic ages and regional metamorphic events of the entire Chinese Altai region, the Mengku deposit is even younger than the youngest major magmatic event 320 Ma ago as well as the youngest Buchan type metamorphic event 290 Ma ago (Fig. 3c). The timing of the regional shear zone movements thus bracket the formation age of the Mengku deposit.

### Meteoric water dominating hydrothermal fluids

Oxygen isotopes of different minerals deposited from hydrothermal fluids can be used to trace their origins. The δ^18^O of the magmatic water that equilibrated with the melt is approximately 5‰–8‰, whereas the δ^18^O of marine carbonate is usually >20‰^28^. If such sources contribute to the fluid, the fluid records the corresponding oxygen isotope characteristics. Marble is exposed at the northeast and southwest of the deposit^24^, which belongs to the Kangbutiebao Formation. If the carbonate precipitated from some isolated fluids in the skarn system, the garnet would record a high-δ^18^O signature, which is inconsistent with our results. The observed δ^18^O values of garnet (5~10‰) in many typical magmatic hydrothermal skarn deposits^29–32^ are similar to those of the magmatic water, clearly indicating that the magmatic water was the primary source of the hydrothermal fluids. However, the δ^18^O of garnet in Mengku is only 1‰, whereas the δ^18^O of hydrothermal zircons are as low as 2.4‰^11^. Because the zircon directly crystallized from a fluid, such low δ^18^O values cannot be explained by a magmatic water origin mixed with limestone wall rock or calcium-rich volcanoclastics^11^. The low δ^18^O of garnet is also inconsistent with a magmatic origin. The combined high-resolution ages of regional magmatic events were at 420~360 Ma and 330~270 Ma, respectively. After ~270 Ma, the Chinese Altai region went into a magmatic lull stage^15^. The 420–360 Ma metamorphic events are interpreted by a transformation from middle-temperature/middle-pressure Barrovian type to low-pressure/high-temperature Buchan type metamorphism during tectonic switching from northwest to southeast shortening events. In the early-middle Permian (290~270 Ma), another high-temperature Buchan type metamorphic event occurred during northeast-southwest shortening of the Chinese Altai Orogen^19,26^. After 270 Ma, no regional metamorphic event occurred. Therefore, neither regional magmatic or metamorphic events could have produced hydrothermal fluids. However, the 255 Ma mineralization is not much later than the youngest Buchan type metamorphism at 270 Ma, which could have increased the regional geothermal gradient over time.

Although various terranes amalgamated together in the early to middle Permian of the Chinese Altai Orogen, thus ceasing magmatism and metamorphism, the terranes continued to move, producing many shear zones along terrane boundaries during the late Permian to Triassic (Fig. 3c)^13,20,33,34^. Peak P-T conditions of the 246 ± 18 Ma garnet-amphibolite schists reached 5.8 ± 1.7 kbar and 643 ± 117 °C^13^, indicating it formed at a crustal depth. A recent study suggests that meteoric water penetrated down to 10 km depth along faults and circulated back up without magmatic driving^35^. According to the P-T constraints of the garnet-amphibolite schist in close proximity to the Mengku deposit, the temperature at ~10 km depth is 290~420 °C. As mentioned above, the Mengku deposit and the garnet-amphibolite schists are located at the hanging wall of the Barils fault and Erqis shear zone. If the Erqis shear zone connects with the Barils thrust at 10 km depth, shear heating can further increase the temperature along the fault by up to 200 °C^36^. The predicted temperature range at 10 km depth is consistent with a range from 300 °C to ~450 °C, which is consistent with models of shear heating^36^. Due to thrusting, meteoric water can be transported efficiently downwards along faults. Temperatures can facilitate local meteoric water circulation that interacts with wall rock, leaching mobile cations^37^. The water/rock ratio can be calculated from the following equation^38^:

$$\begin{array}{rcl}{{\rm{\delta }}}^{{\rm{18}}}{{\rm{O}}}_{{\rm{hydrothemal}}{\rm{fluid}}} & = & {({\rm{\delta }}}^{{\rm{18}}}{{\rm{O}}}_{{\rm{orginal}}{\rm{rock}}}+{\mathrm{water}/\mathrm{rock}{\rm{\delta }}}^{{\rm{18}}}{{\rm{O}}}_{{\rm{orginal}}{\rm{water}}}\\  &  & -{{\rm{\Delta }}}^{{\rm{18}}}{{\rm{O}}}_{{\rm{orginal}}{\rm{rock}}-{\rm{water}}})/(1+\mathrm{water}/\mathrm{rock})\end{array}$$

**Figure 4:**
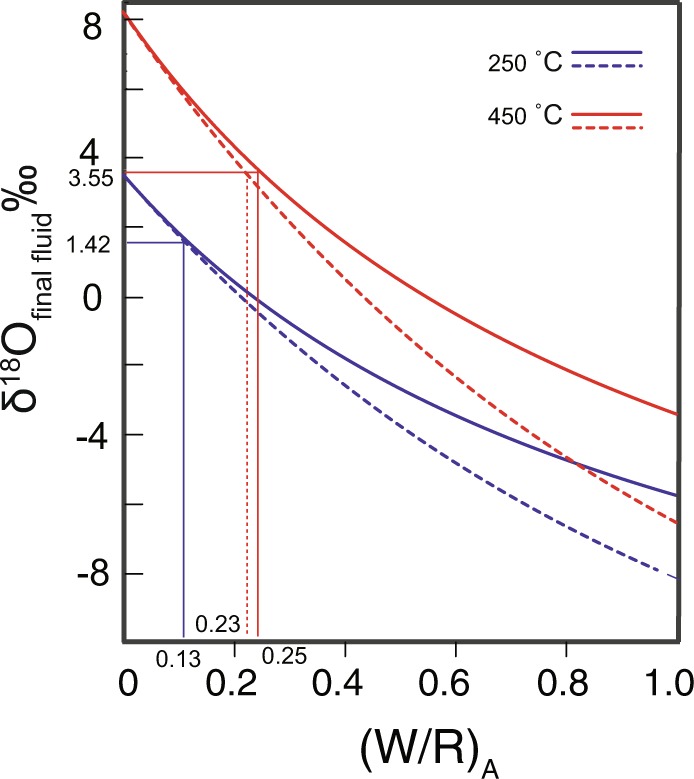
(**a**) Oxygen isotopic compositions of fluid and rock reacted with volcanoclastic rocks and meteoric water. Dashed and solid lines for an open and closed system respectively. The figure illustrates that reaction results in the fluid to crystalize garnet with δ^18^O of 1‰ at associated water-rock ratios under a temperature of 250~450 °C. The marked (W/R)_A_ from 0.13, 0.23, and 0.25 are water/rock atomic ratios, and the calculated water/rock mass ratio are 6.5%, 11.5%, and 12.5%. The detailed calculation procedures could be found in the Supplementary Dataset 2.

- The hydrothermal temperature (~250~450 °C) is based on the homogenous temperature of fluid inclusions in garnet^23^. The δ^18^O of hydrothermal fluids crystalizing the garnet with δ^18^O_garnet_ of 1‰ under temperatures at 250~450 °C is 1.42~3.55‰.
- The end members of rock δ^18^O values can be calculated from zircon δ^18^O values based on equations of δ^18^O_whole rock_–δ^18^O_zircon_ = 0.0612 (wt.% SiO_2_) + 2.5‰. As reported by Wan *et al*.^11^, the δ^18^O zircon of volcanoclastic host rock is 7.0~8.0‰, hence the δ^18^O_whole rock_ (78% SiO_2_) should be ~10‰.
- The end members of original water δ^18^O values in the Mengku region from δD-δ^18^O isotopic studies of garnet in the deposit (Supplementary Dataset 2)^23^ can also be calculated. δD_V-SMOW_ and δ^18^O_V-SMOW_ of the Mengku garnet is −85~−127‰, and 0.2~2.1‰, respectively^23^. The δD value of meteoric water would be relatively constant during the water-rock reaction because of low H abundances in the rock. δD and δ^18^O in meteoric global water have a linear relationship δD = 8δ^18^O + 10^39^. According to the reported δD, the calculated δ^18^O of meteoric water in the Mengku region should be −15 ± 2‰^23^.
- The Δ^18^O_orginalrock-water_ can be calculated by 1000Inα = 2.68 × (10^6^/(homogenous temperature + 273)^2^)–3.29 by known lnα^28^. According to the above constraints, the water/rock mass ratios under temperatures of 250~450 °C are approximately 0.065–0.115 in an open system and 0.065–0.125 in a closed system (Fig. 4). The open system may be more relevant in this study, with the shear zone acting as a conduit and adding meteoric water by hydrothermal fluids.

### Implication for mineralization

**Figure 5:**
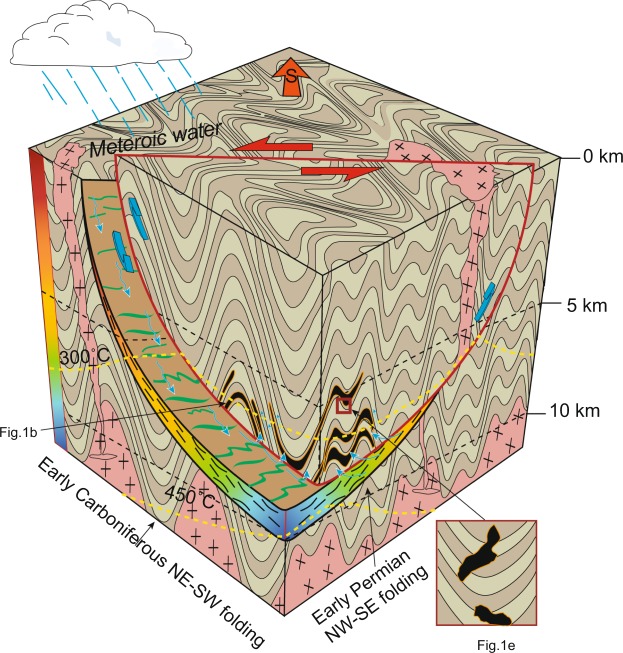
A conceptual model of the Mengku deposit. The model illustrates the shear movement and thrust motion circulating the meteoric water. Skarn iron ore layers follow with fold strata as in the Fig. 1b. The deformation styles and ages of the two stages of deformation of strata are from^19,26^. The inset corresponding to the Fig. 1e represents the skarn that cross-cuts the fold. The green lines on the shear zone indicate the shear direction. The pink rock is granitic basement. The color bar on the left is the relative water content the shear zone on a temperature color scale. The temperature of the shear zone increases and the water content decreases with the depth. The isotherm refers to Leloup *et al*.^36^.

In this study, the LA-ICP-MS U-Pb garnet age (255 Ma) of the Mengku deposit is consistent with regional shear-thrust movements (246 Ma) and is much younger than the magmatic-metamorphic events recorded both in the ore deposits (378 Ma) and the Chinese Altai region (270 Ma). We therefore model the chronological course of events from mineralization to shear movement using the direct U-Pb dates on garnet and hydrothermal zircons from ores and the activation timing of regional shear systems (Fig. 5). We further interpret that the δ^18^O_garnet_ is only 1‰, indicating a meteoric water-rich signature. The δ^18^O_garnet_ resulted from water-rock reactions with water/rock mass ratios of 0.065–0.115 in an open system from 250–450 °C, with 6.5–11.5% of water in the region. Previous studies identified rock permeability increasing along fracture zones in the crust^40^. Hence, such water fractions are common in the region along the fault at 10 km depths^41,42^. Ductile deformation occurs in quartz at ~10 km, and feldspar exhibits brittle structure^43^. Due to the brittle deformation of feldspar, there would be small passages in the shear zone in which fluids could be channeled. Additionally, meteoric water in shear-thrust movements in the Chinese Altai Orogen efficiently penetrate the fault at ~10 km depths, which is also reported in other fault zones^35,44^. Higher temperatures occur at depth in the shear zone that are higher than the regional geothermal gradient because of the additional shear heat^36^. According to the regional geothermal gradient and thrust-shear movements, we propose that the faults provided conduits and heat ‘engines’ for the circulation of fluids along the hanging wall rocks. The wall rock probably retains ore-forming elements such as Fe and Ca. The skarn mineral assemblage formed by silica- and calcium-rich deposits induced by shear-thrust movement is different to regular skarn ores deposited by the reaction between magmatic-derived hydrothermal fluids and calcium-rich components^45^_._ Notably, many different kinds of ore deposits have been reported in the adjunct region of regional shear zones in orogenic belts, and their mineralization times occurred coeval with active shear zones rather than around the time of regional magmatism, e.g., the Lupin gold deposit in Canada and the Tieluping silver deposit in China^46–51^. Such shear zones could help the rock to release and circulate fluids in an orogenic belt, and thus must play essential roles as both fluid conduits and heat sources^40^. Our model of hydrothermal gradients along shear zones circulating fluids during shear movement that lead to mineralized ores should be of significant economic relevance for future mineral exploration. Because shear zones are ubiquitous and widespread during geological history, their role in economic geology may have been heretofore overlooked.

## Methods

### Sample preparation

Garnet, zircon and meta-rhyolitic rocks in this study were sampled in the vicinity of the No. 7 orebody. The garnet is large enough to collect by hand, and zircons were separated by a combination of heavy liquid and magnetic techniques. For U-Pb and major element analytical purpose, garnet and zircon were mounted in epoxy resin separately and polished to remove the upper one-third of the grains. Cathodoluminescence (CL) images were obtained in order to identify internal structures and choose potential target sites for U-Pb analyses.

### Major oxides of garnet by JEOL JXA-8100 electron microprobe and major-element oxides of rock by XRF

They were determined at the Institute of Geology and Geophysics, Chinese Academy of Sciences (IGGCAS). Analyses were undertaken to employ a 1–2 μm beam spot size, with 15 kV accelerating voltage, counting time of 20 s and 20 nA beam current per element. A program based on the ZAF procedure was used for data correction. Major-element oxides were analyzed on fused glass disks employing a Phillips PW 1500 × -ray fluorescence spectrometer. The precision and accuracy of the major-element data as determined with the Chinese whole-rock granite standard GSR-1 are ≤5% and ca. 5% (2 s), respectively.

### Garnet U-Pb analyses by LA-ICP-MS

Isotopic measurements were carried out at an Agilent 7500a (Agilent Technologies, Japan) mass spectrometer coupled with an excimer 193 nm laser ablation system (Geolas 2005, Lambda Physic, Gottingen, Germany) at the IGGCAS. Detailed analytical procedures and experimental parameters are described by Seman *et al*.^9^ and Yang *et al*.^52^. Laser working conditions were: 50 s ablation time, 6 Hz repetition rate, 7 mJ energy with 90% beam attenuation, resulting in a fluence of 10 J cm^−2^. He carrier gas flow rate and Ar sample gas flow rate were set at 0.67 L/min and 1.05 L/min, respectively. Each spot is 60 μm in diameter, which consists of an approximately 25 s background acquisition and 60 s specimen data acquisition. During routine analysis, background intensities were measured on-peak with laser off for the initial 25 s, and the laser was fired onto the samples for 60 s. Standards were two matrix-matched external references andradite (Willsboro and Magana Mali) and one internal standard NIST 612 glass. ^207^Pb/^206^Pb and ^206^Pb/^238^U ratios were calculated using GLITTER 4.0^53^. Data reduction was carried out using the IsoplotR program^54^.

### Zircon U-Pb analyses by SIMS

Measurements of U, Th, and Pb isotopes of zircon were conducted using a Cameca-IMS 1280 large-radius Secondary Ion Mass Spectrometry (SIMS) at IGGCAS. Zircon U-Th-Pb ratios and absolute abundances were determined relative to the standard zircon 91500^55^, analyses of which were interspersed with those of unknown grains, using operating and data processing procedures similar to those described by Li X. *et al*.^56^. A long-term uncertainty of 1.5% (1 RSD) for ^206^Pb/^238^U measurements of the standard zircons was propagated to the unknowns of Li Q. *et al*.^57^, although the measured ^206^Pb/^238^U error in a specific session is generally around 1% (1 RSD) or less. Measured compositions were corrected for common Pb using non-radiogenic ^204^Pb. Corrections are sufficiently small to be insensitive to the choice of common Pb composition, and an average of present-day crustal composition^58^ is used for the common Pb assuming that the common Pb is mostly surface contamination introduced during sample preparation.

### Garnet O analyses by stable isotopes mass spectrometer

Oxygen was extracted from garnet by the BrF method^59^. The analytical precisions were ±0.1‰. Garnet oxygen compositions were determined on a Finnigan-MAT252 mass spectrometer at IGGCAS.

## Supplementary information

Supplementary Dataset1-3

## References

[1] Newton, R. C. & Perkins, D. Thermodynamic calibration of geobarometers based on the assemblages garnet-plagioclase-orthopyroxene (clinopyroxene)-quartz. *American Mineralogist* **67**, 203–222, 10.1.1.566.2455 (1982).

[2] Harley SL, Green DH (1982). Garnet–orthopyroxene barometry for granulites and peridotites. Nature.

[3] Jamtveit B, Hervig RL (1994). Constraints on Transport and Kinetics in Hydrothermal Systems from Zoned Garnet Crystals. Science.

[4] Becker H (1993). Garnet peridotite and eclogite Sm-Nd mineral ages from the Lepontine dome (Swiss Alps): New evidence for Eocene high-pressure metamorphism in the central Alps. Geology.

[5] Duchene S (1997). The Lu-Hf dating of garnets and the ages of the Alpine high-pressure metamorphism. Nature.

[6] Meinert, L. D., Nicolescu, S., Mortensen, J. K. & Cornell, D. H. In GSA Annual Meeting (Boston, Mass, 2001).

[7] Deng X-D, Li J-W, Luo T, Wang H-Q (2017). Dating magmatic and hydrothermal processes using andradite-rich garnet U–Pb geochronometry. Contributions to Mineralogy and Petrology.

[8] Wafforn S (2018). Andradite garnet U-Pb geochronology of the Big Gossan skarn, Ertsberg-Grasberg mining district, Indonesia. Economic Geology.

[9] Seman S, Stockli DF, McLean NM (2017). U-Pb geochronology of grossular-andradite garnet. Chemical Geology.

[10] Zhang Y (2018). Dating Ore Deposit Using Garnet U–Pb Geochronology: Example from the Xinqiao Cu–S–Fe–Au Deposit, Eastern China. Minerals.

[11] Wan B, Xiao W, Zhang L, Han C (2012). Iron mineralization associated with a major strike-slip shear zone: Radiometric and oxygen isotope evidence from the Mengku deposit, NW China. Ore Geology Reviews.

[12] Windley BF (2002). Neoproterozoic to Paleozoic geology of the Altai Orogen, NW China: new zircon age data and tectonic evolution. Journal of Geology.

[13] Briggs SM, Yin A, Manning CE, Chen ZL, Wang XF (2009). Tectonic development of the southern Chinese Altai Range as determined by structural geology, thermobarometry, ^40^Ar/^39^Ar thermochronology, and Th/Pb ion-microprobe monazite geochronology. Geological Society of America Bulletin.

[14] Cai K, Sun M, Yuan C, Long X, Xiao W (2011). Geological framework and Paleozoic tectonic history of the Chinese Altai, NW China: a review. Russian Geology and Geophysics.

[15] Zhang J (2017). Tracking deep ancient crustal components by xenocrystic/inherited zircons of Palaeozoic felsic igneous rocks from the Altai–East Junggar terrane and adjacent regions, western Central Asian Orogenic Belt and its tectonic significance. International Geology Review.

[16] Broussolle A (2018). Polycyclic Palaeozoic evolution of accretionary orogenic wedge in the southern Chinese Altai: Evidence from structural relationships and U Pb geochronology. Lithos.

[17] Jiang Y (2019). Structural and Geochronological Constraints on Devonian Suprasubduction Tectonic Switching and Permian Collisional Dynamics in the Chinese Altai, Central. Asia. Tectonics.

[18] Wan B, Zhang L, Xiao W (2010). Geological and geochemical characteristics and ore genesis of the Keketale VMS Pb-Zn deposit, Southern Altai metallogenic belt, NW China. Ore Geology Reviews.

[19] Zhang J (2015). Distinct deformational history of two contrasting tectonic domains in the Chinese Altai: Their significance in understanding accretionary orogenic process. Journal of Structural Geology.

[20] Laurent-Charvet Sébastien, Charvet Jacques, Monié Patrick, Shu Liangshu (2003). Late Paleozoic strike-slip shear zones in eastern central Asia (NW China): New structural and geochronological data. Tectonics.

[21] Wang, Y. W., Wang, J. B., Wang, S. L., Ding, R. F. & Wang, L. J. In Tectonic Evolution and Metallogeny of the Chinese Altay and Tianshan Vol. Proceeding Volume of the International Symposium of the IGCP-473 Project in Urumqi (eds Mao, J. W *et al*.) 181–200 (Centre for Russian and Central Asian Mineral Studies, Natural History Museum, 2003).

[22] Xu L, Mao J, Yang F, Zheng J (2010). Geology, geochemistry and age constraints on the Mengku skarn iron deposit in Xinjiang Altay, NW China. Journal of Asian Earth Sciences.

[23] Yang, F. Q. *et al*. Ore-forming fluids and metallogenesis of Mengku iron deposit in Altay, Xinjiang. *Mineral Deposits* 27, 659–678 (in Chinese with English abstract) (2008).

[24] Yang F (2010). Geochronology and geochemistry of the granites from the Mengku iron deposit, Altay Mountains, northwest China: implications for its tectonic setting and metallogenesis. Australian Journal of Earth Sciences.

[25] Tong Y (2014). Post-accretionary Permian granitoids in the Chinese Altai orogen: Geochronology, petrogenesis and tectonic implications. American Journal of Science.

[26] Broussolle A (2019). Are the Chinese Altai “terranes” the result of juxtaposition of different crustal levels during Late Devonian and Permian orogenesis?. Gondwana Research.

[27] Zhai, Y., Yao, S. & Cai, K. Mineral Deposits (3rd Edition) 413 (in Chinese) (Geological Publishing House, 2011).

[28] Taylor BE (1986). Magmatic volatiles; isotopic variation of C. H, and S. Reviews in Mineralogy and Geochemistry.

[29] Belley P (2017). Origin Of Scapolite-Hosted Sapphire (Corundum) Near Kimmirut, Baffin Island, Nunavut, Canada. The Canadian Mineralogist.

[30] Rose, A., Herrick, D. & Deines, P. An oxygen and sulfur isotope study of skarn-type magnetite deposits of the Cornwall type, southeastern Pennsylvania. **80** (1985).

[31] Xu J, Zheng Y, Sun X, Shen Y (2016). Alteration and mineralization at the Zhibula Cu skarn deposit, Gangdese belt, Tibet. Ore Geology Reviews.

[32] Meinert LD, Hedenquist JW, Satoh H, Matsuhisa Y (2003). Formation of Anhydrous and Hydrous Skarn in Cu-Au Ore Deposits by Magmatic Fluids. Economic Geology.

[33] Wan B, Xiao W, Windley BF, Yuan C (2013). Permian hornblende gabbros in the Chinese Altai from a subduction-related hydrous parent magma, not from the Tarim mantle plume. Lithosphere.

[34] Xiao W (2009). Paleozoic multiple subduction-accretion processes of the southern Altaids. American Journal of Science.

[35] Diamond LW, Waber HN, Wanner C (2018). Penetration depth of meteoric water in orogenic geothermal systems. Geology.

[36] Leloup PH, Ricard Y, Battaglia J, Lacassin R (1999). Shear heating in continental strike-slip shear zones:model and field examples. Geophysical Journal International.

[37] Lasaga AC (1984). Chemical kinetics of water-rock interactions. Journal of Geophysical Research: Solid Earth.

[38] Stakes DS, O’Neil JR (1982). Mineralogy and stable isotope geochemistry of hydrothermally altered oceanic rocks. Earth and Planetary Science Letters.

[39] Craig H (1961). Isotopic Variations in Meteoric Waters. Science.

[40] Boutoux A (2014). Fluid systems above basement shear zones during inversion of pre-orogenic sedimentary basins (External Crystalline Massifs, Western Alps). Lithos.

[41] Hyndman R, Shearer P (1989). Water in the lower continental crust: modelling magnetotelluric and seismic reflection results. Geophysical Journal International.

[42] Zharikov AV, Vitovtova VM, Shmonov VM, Grafchikov AA (2003). Permeability of the rocks from the Kola superdeep borehole at high temperature and pressure: implication to fluid dynamics in the continental crust. Tectonophysics.

[43] Fossen H, Cavalcante GCG (2017). Shear zones – A review. Earth-Sci. Rev..

[44] deMartin BJ, Sohn RA, Pablo Canales J, Humphris SE (2007). Kinematics and geometry of active detachment faulting beneath the Trans-Atlantic Geotraverse (TAG) hydrothermal field on the Mid-Atlantic Ridge. Geology.

[45] Meinert, L. D., Dipple, G. M. & Nicolescu, S. In Economic Geology 100th Anniversary Volume (eds Hedenquist, J. W., Thompson, J. F. H., Goldfarb, R. J. & Richards, J. P) 299–405 (Littleton, CO, Society of Economic Geologists, 2005).

[46] Groves DI, Goldfarb RJ, Robert F, Hart CJR (2003). Gold deposits in metamorphic belts: overview of current understanding,outstanding problems, future research, and exploration significance. Economic Geology.

[47] Goldfarb, R. J. *et al*. In Economic Geology 100th Anniversary Volume (eds Hedenquist, J. W., Thompson, J. F. H., Goldfarb, R. J., Richards, J. P.) 407–450 (Society of Economic Geologists, 2005).

[48] Pirajno, F. Hydrothermal Processes and Mineral System. 1250 (Springer Press, 2009).

[49] Bullis HR, Hureau RA, Penner BD (1994). Distribution of gold and sulfides at Lupin, Northwest Territories. Economic Geology.

[50] Pan Y, Fleet ME, Stone WE (1991). Skarn mineralization (Cr, Fe, Au) in an Archean greenstone belt, White River Property, Hemlo area, Ontario. Economic Geology.

[51] Chen YJ, Pirajno F, Sui YH (2004). Isotope geochemistry of the Tieluping silver-lead deposit, Henan, China: A case study of orogenic silver-dominated deposits and related tectonic setting. Mineralium Deposita.

[52] Yang Y-H (2018). U–Pb age determination of schorlomite garnet by laser ablation inductively coupled plasma mass spectrometry. Journal of Analytical Atomic Spectrometry.

[53] Griffin, W. L., Powell, W. J., Pearson, N. J. & O Reilly, S. Y. In Laser Ablation-ICP MS in the Earth Sciences: Current Practices and Outstanding Issues Vol. Mineralogical Association of. Canada Short Course 40 (ed Sylvester, P. J.) 308–311 (2008).

[54] Vermeesch P (2018). IR: A free and open toolbox for geochronology. Geoscience Frontiers.

[55] Wiedenbeck M (1995). Three natural zircon standards for U-Th-Pb, Lu-Hf, trace element and REE analyses. Geostandards Newsletter.

[56] Li X (2009). Precise determination of Phanerozoic zircon Pb/Pb age by multicollector SIMS without external standardization. Geochemistry. Geophysics. Geosystems.

[57] Li QL (2010). Precise U–Pb and Pb–Pb dating of Phanerozoic baddeleyite by SIMS with oxygen flooding technique. Journal of Analytical Atomic Spectrometry.

[58] Stacey JS, Kramers JD (1975). Approximation of terrestrial lead isotope evolution by a two-stage model. Earth and Planetary Science Letters.

[59] Clayton RN, Mayeda TK (1963). The use of bromine pentafluoride in the extraction of oxygen from oxides and silicates for isotopic analysis. Geochimica et Cosmochimica Acta.

